# Association of hypoglycemic symptoms with patients' rating of their health-related quality of life state: a cross sectional study

**DOI:** 10.1186/1477-7525-8-86

**Published:** 2010-08-19

**Authors:** Fernando Alvarez-Guisasola, Donald D Yin, Gonzalo Nocea, Ying Qiu, Panagiotis Mavros

**Affiliations:** 1 Centro de Salud La Calzada II, Gijon, Spain; 2 Outcomes Research, Merck & Co., Inc., Whitehouse Station, NJ, USA; 3 Outcomes Research, MSD, Madrid, Spain

## Abstract

**Background:**

To evaluate the association between patient-reported hypoglycemic symptoms with ratings of their health-related quality of life state and patient-reported adverse events in patients with type 2 diabetes mellitus (T2DM).

**Methods:**

This observational, multicenter, cross sectional study was based on a sample of patients with T2DM from seven European countries who added sulfonylurea or thiazolidinedione to metformin monotherapy between January 2001 and January 2006. Included patients were required to have at least one hemoglobin A_1c _(HbA_1c_) measurement in the 12 months before enrollment and to not be receiving insulin. Demographic and clinical data from medical records were collected using case report forms. Questionnaires measured patient-reported hypoglycemic symptoms, health-related quality of life (EuroQol visual analogue scale, EQ-5D VAS), and treatment-related adverse events.

**Results:**

A total of 1,709 patients were included in the study. Mean patient age was 63 years, 45% were female, mean HbA_1c _was 7.06%, and 28% were at HbA_1c _goal (HbA_1c _< 6.5%). Hypoglycemic symptoms during the 12 months before enrollment were reported by 38% of patients; among whom 68% reported their most severe symptoms were mild, 27% moderate, and 5% severe. Adjusted linear regression analyses revealed that patients reporting hypoglycemic symptoms had significantly lower EQ-5D VAS scores indicating worse patient-reported quality of life (mean difference -4.33, p < 0.0001). Relative to those not reporting symptoms, the adjusted decrement to quality of life increased with greater hypoglycemic symptom severity (mild: -2.68, *p *= 0.0039; moderate: -6.42, *p *< 0.0001; severe: -16.09, *p *< 0.0001). Patients with hypoglycemia reported significantly higher rates of shakiness, sweating, excessive fatigue, drowsiness, inability to concentrate, dizziness, hunger, asthenia, and headache (*p *< 0.0001 for each comparison).

**Conclusions:**

Hypoglycemic symptoms and symptom severity have an adverse effect on patients' rating of their health related quality of life state. Hypoglycemic symptoms are correlated with treatment-related adverse effects. Minimizing the risk and severity of hypoglycemia may improve patients' quality of life and clinical outcomes. Results are subject to limitations associated with observational studies including the potential biases due to unobserved patient heterogeneity and the use of a convenience sample of patients.

## Background

Hypoglycemia is a common complication of diabetes management that may adversely impact clinical outcomes. Although improved glycemic control reduces the risks of macrovascular and microvascular complications, treatment aimed toward increasingly stringent, consensus-guided glycemic targets may be associated with hypoglycemia [1-3]. While the risk of hypoglycemia is particularly elevated in patients receiving insulin therapy, patients with type 2 diabetes mellitus (T2DM) treated with insulin secretagogues (e.g., sulfonylureas, meglitinides) are also at increased risk of experiencing hypoglycemic symptoms [4-7]. The UKPDS reported that 31% of patients on first generation sulfonylureas (glibenclamide) experienced mild hypoglycemic symptoms during the first year of the study follow-up [8]. Third-generation sulfonylureas (e.g., glimepiride, glipizide, and gliclazide) and the metiglinides (e.g., repaglinide and nateglinide) seem to be associated with lower rates of hypoglycemia [9]. According to the ADOPT study, patients on insulin sensitizers (metformin or rosiglitazone) experienced hypoglycemia at a rate of about 10% over 5 years of treatment [10].

Previous work by other groups has demonstrated that hypoglycemia is inversely related to quality of life (QOL) and well-being in patients with T2DM [11]. Patients with hypoglycemia tended to have a lower utility score from questions of the EuroQol-5D (EQ-5D), a standardized measure of health-related QOL (HRQOL) [11]. Other studies have demonstrated an inverse association between hypoglycemia and QOL according to the Quality of Well-Being Self-Administered questionnaire, as well as the EQ-5D and the short form-36 (SF-36) [12-14]. However, data on self-reported HRQOL specifically in patients with T2DM that are suboptimally managed using metformin are limited. Accordingly, the aim of the present study was to evaluate the impact of patient reported hypoglycemic symptoms on ratings of their health-related QOL state in patients with T2DM receiving oral antihyperglycemic treatment in usual-care clinical settings across several European countries, using the EQ-5D visual analogue scale (VAS).

## Methods

### Study description

The Real-Life Effectiveness and Care Patterns of Diabetes Management (RECAP-DM) study was a European multicenter observational study involving patients with T2DM on oral antihyperglycemic treatments. The study was conducted in endocrinology, diabetology, and general-practice clinics and physician offices in Finland, France, Germany, Norway, Poland, Spain, and the United Kingdom. Study centers were selected randomly from a list comprising a convenience sample of physicians from each country.

Eligible patients were identified for participation in the study during the enrollment period, from June 2006 to February 2007. Criteria for study eligibility were ages ≥ 30 years, diagnosis with T2DM as defined by the American Diabetes Association, addition of a sulfonylurea or thiazolidinedione to metformin monotherapy on a date (index date) from January 2001 to January 2006, and at least one hemoglobin A_1c _(HbA_1c_) measurement in the 12-month period before the enrollment date [15]. Excluded were patients with T1DM; pregnant women, including those with gestational diabetes; diabetes secondary to other factors (e.g., malnutrition, infection, surgery); and those who could not complete questionnaires or were participating in another clinical study. All participating patients were asked to sign an informed-consent form prior to enrollment. Both the informed-consent document and study protocol were reviewed and approved by local ethical review boards in each country.

### Study measurements

Case report forms were used to collect patient demographic and clinical data from medical records. These included patient age and sex, smoking status, alcohol use, physical activity, body mass index, history of microvascular events (blindness, renal failure, or amputation) and cardiovascular events (ischemic heart disease, congestive heart failure, myocardial infarction, stroke, atrial fibrillation, peripheral vascular disease) during the observation period prior to the enrollment date, time since diabetes diagnosis, most recent HbA_1c _measurement within the year before the enrollment date, and whether patients were at HbA_1c _goal. Adequate glycemic control (at goal) was defined according to the International Diabetes Federation as HbA_1c _< 6.5%, where HbA_1c _refers to the most recent measurement in the 12 months before enrollment [16].

### Study questionnaires

A patient questionnaire was used to solicit data on patients' reported experiences of hypoglycemic symptoms. Patients' experiences of hypoglycemic symptoms were based on their answers to the question "Have you ever felt symptoms of hypoglycemia (low blood sugar) in the last year?" Patients rated their hypoglycemic symptom severity by selecting one of the following response options: (1) "little or no interruption of your activities, and you didn't feel you needed assistance to manage symptoms" (mild); (2) "some interruption of your activities, but you didn't feel you needed assistance to manage symptoms" (moderate); (3) "you felt you needed assistance of others to manage symptoms (e.g., to bring you food or drink)," (severe); or (4) "you needed medical attention (e.g., called an ambulance, visited an emergency room or hospital, or saw a doctor or nurse)" (very severe). Severe and very severe symptoms were consolidated and referred to as "severe." Symptom severity was classified according to the most severe symptom reported.

Patient-reported HRQOL was evaluated using the EQ-5D VAS, a brief, standardized, generic measure of HRQOL that provides a profile of patient function and a global health state rating [17]. EQ-5D VAS records the respondent's self-rated health status on a graduated (0-100 mm) scale, with higher scores for higher HRQOL [18,19]. This provides a direct valuation of the respondent's current state of health.

Finally, treatment-related adverse events were evaluated by providing patients with a list of potential adverse events including excessive fatigue, drowsiness, inability to concentrate, dizziness, sweating, hunger, shakiness, asthenia, and headache. Patients were asked to record how much they were bothered by these adverse events on a scale from 'did not experience' to 'extremely bothered'. A dichotomous response ('did not experience' vs. 'bothered') was analyzed in relation to different levels of hypoglycemic symptom severity.

### Statistical analyses

The hypothesis of no association of hypoglycemic symptoms with measures of QOL was examined using the *t *test. The F test was used to test the null hypothesis of no association of hypoglycemic symptom severity with QOL. The chi-square test was used to test the null hypothesis of no association between the experience of each adverse event and hypoglycemic symptoms. Statistical significance was evaluated at α = 0.05.

Adjusted linear regression models were used to examine the association between hypoglycemic symptoms and symptom severity with patient QOL (EQ-5D VAS) after adjusting for other predictors. The reported regressions are the result of a backward selection model technique applied on a model including all variables that were significant at *p *≤ 0.20 in univariate analysis except for symptom severity indicators (first model) or hypoglycemic symptoms indicator (second model).

## Results

Of 2,146 patients recruited to the study, 2,139 completed the study surveys at the enrollment date, and 2,052 also satisfied inclusion and exclusion criteria. After excluding patients who used insulin prior to the enrollment date and patients without an HbA_1c _test measurement during the 12 months prior to the enrollment date, the final sample consisted of 1,709 patients. Most patients were recruited in 2006 (*N *= 972) or 2007 (*N *= 725), and most were recruited in Spain (25.8%), the United Kingdom (20.0%), or Germany (19.1%). Mean (SD) patient age was 62.94 (10.58) years and 45% were female (Table 1). Mean (SD) HbA_1c _was 7.06 (1.06), and 28% of patients were at the HbA_1c _goal of < 6.5%.

**Table 1:** Patient demographic and clinical characteristics

Characteristic	All Patients (*N *= 1709)	Spain (*N *= 441)	France (*N *= 150)	UK (*N *= 342)	*Norway* (*N *= 48)	Finland (*N *= 162)	Germany (*N *= 326)	Poland (*N *= 240)
Age (years)	62.94 ± 10.58	63.21 ± 10.52	62.96 ± 10.06	62.80 ± 11.80	62.25 ± 9.57	61.74 ± 10.08	64.90 ± 10.21	60.93 ± 9.78
Female (%)	45.08	45.58	34.00	40.76	45.83	48.15	48.00	51.05
Current Smokers (%)	13.53	14.09	16.11	14.84	9.09	11.73	9.09	17.35
No Alcohol Use (%)	29.37	49.04	24.67	28.27	20.51	27.78	18.44	14.56
No Regular Physical Activity(%)	35.38	29.50	40.67	41.43	38.10	19.14	44.20	32.71
Physical Activity 3-5 Times/Week (%)	24.92	29.98	13.33	11.53	21.43	53.09	14.11	38.79
Body Mass Index (kg/m^2^)	31.7 ± 6.8	32.1 ± 7.5	31.0 ± 5.0	31.1 ± 6.9	30.8 ± 4.3	32.7 ± 5.6	31.6 ± 5.7	31.53 ± 8.3
Microvascular Events (%)	2.16	2.28	0.67	3.23	2.08	3.11	0.31	3.33
Cardiovascular Events (%)	26.39	18.68	26.00	30.79	22.92	25.47	25.39	38.25
Duration of T2DM (years)	7.84 ± 5.08	8.25 ± 5.13	8.43 ± 5.26	7.05 ± 4.95	8.19 ± 5.50	7.07 ± 3.95	9.15 ± 5.12	6.46 ± 5.03
HbA_1c _(%)*	7.06 ± 1.06	7.05 ± 1.20	7.00 ± 1.00	7.22 ± 1.07	7.31 ± 1.06	6.99 ± 1.05	7.02 ± 0.99	6.89 ± 0.88
Patients with HbA_1c _< 6.5% (%)	27.91	31.75	30.67	19.30	16.67	34.57	26.99	30.42

*Refers to the most recent HbA_1c _measurement within the 12 months prior to visit

Data are mean ± SD unless otherwise specified

T2DM = type 2 diabetes mellitus; HbA_1c _= hemoglobin A_1c_

**Figure 1:**
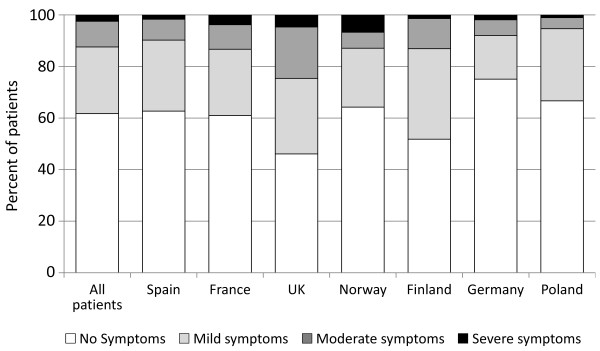
Prevalence of patient-reported experience of hypoglycemic symptoms* and symptom severity^† ^during the 12 months prior to the patient enrollment date. * Hypoglycemic symptoms are based on the response to the question: "Have you ever felt symptoms of hypoglycemia (low blood sugar) in the last year?". † Hypoglycemic symptom severity is based on the most severe form reported by a patient, i.e. if a patient reports both 'mild' and 'moderate' symptoms, the patient is listed only under the 'moderate' symptoms group. Mild symptoms were defined as: Little or no interruption of your activities, and you didn't feel you needed assistance to manage symptoms. Moderate symptoms were defined as: Some interruption of your activities, but you didn't feel you needed assistance to manage symptoms. The severe symptoms group is a consolidation of the 'severe' and 'very severe' symptoms that were respectively defined as: Felt that you needed assistance of others to manage symptoms (for example, to bring you food or drink), and needed medical attention (for example, called an ambulance, visited an emergency room or hospital, or saw a doctor or nurse).

Hypoglycemic symptoms, during the 12 months prior to the enrollment date, were reported by 38.4% of patients, with the prevalence of symptoms ranging from 24.2% in Germany to 53.6% in the United Kingdom (Figure 1). Among patients reporting hypoglycemic symptoms, 68.1% reported that their symptoms were mild, 26.8% moderate, and 5.1% severe.

The mean (SD) EQ-5D VAS score was 71.64 (16.44) and was consistent for patients in different countries (Table 2). Patients with hypoglycemic symptoms had significantly lower EQ-5D VAS scores than patients without hypoglycemic symptoms (68.70 [16.58] vs. 73.47 [16.11] respectively), indicating a 4.78 (16.29) decrement of hypoglycemia on patient-reported QOL (*p *< 0.0001). There was an inverse relationship between hypoglycemic symptom severity and patient-reported QOL, with patients reporting severe symptoms having a mean (SD) score of 54.30 (18.77), compared with 65.80 (16.92) for moderate and 70.93 (15.56) for mild symptom severity, and 73.47 (16.11) for patients reporting no symptoms (*p *< 0.0001).

**Table 2:** Patients' rating of their health-related quality of life state (EuroQol Visual Analogue Scale) by experience of hypoglycemic symptoms and hypoglycemic symptom severity - overall and by country

Patient Group	All Patients (*N *= 1709)	Spain (*N *= 441)	France (*N *= 150)	UK (*N *= 342)	Norway (*N *= 48)	Finland (*N *= 162)	Germany (*N *= 326)	Poland (*N *= 240)
All Patients	71.64 ± 16.44	70.42 ± 14.68	71.89 ± 15.51	68.77 ± 17.87	70.15 ± 17.75	70.53 ± 15.91	73.08 ± 16.22	76.80 ± 17.10
**By Experience of Hypoglycemic Symptoms**								
with symptoms	68.70 ± 16.58	68.96 ± 14.09	71.37 ± 13.53	65.35 ± 18.28	75.00 ± 19.20	69.61 ± 17.32	68.27 ± 18.59	72.03 ± 14.61
without symptoms	73.47 ± 16.11	71.26 ± 14.91	72.22 ± 16.72	72.50 ± 16.73	67.48 ± 16.62	71.56 ± 14.53	74.61 ± 15.11	79.14 ± 17.78
VAS score differences	4.78 ± 16.29	2.31 ± 14.62	0.86 ± 15.56	7.16 ± 17.58	-7.52 ± 17.56	1.95 ± 15.94	6.34 ± 16.01	7.12 ± 16.81
*p*-value*	< 0.0001	0.1177	0.7461	0.0003	0.1629	0.4418	0.0072	0.0023
**By Hypoglycemic Symptom Severity**^**†**^								
witout symptoms	73.47 ± 16.11	71.26 ± 14.91	72.22 ± 16.72	72.50 ± 16.73	67.48 ± 16.62	71.56 ± 14.53	74.61 ± 15.11	79.14 ± 17.78
with mild symptoms	70.93 ± 15.56	69.97 ± 14.30	73.73 ± 12.42	67.69 ± 17.75	79.55 ± 9.34	70.07 ± 17.85	73.57 ± 15.01	72.94 ± 14.15
with moderate symptoms	65.80 ± 16.92	66.14 ± 12.78	71.00 ± 13.52	64.65 ± 17.42	90.00 ± 17.32	67.95 ± 16.95	59.95 ± 20.97	65.73 ± 16.94
with severe symptoms	54.30 ± 18.77	65.00 ± 17.68	55.00 ± 12.25	51.54 ± 21.51	43.33 ± 16.07	72.50 ± 3.54	41.75 ± 10.90	80.00 ± 0.00
*p*-value^‡^	< 0.0001	0.1980	0.0866	< 0.0001	0.0010	0.8243	< 0.0001	0.0106

Data are mean ± SD unless otherwise specified

* Based on the *t *test of the null of no differences in the mean quality of life scores between patients with and without hypoglycemic symptoms

^† ^Symptom severity is based on the most severe form reported by a patient, i.e. if a patient reports both 'mild' and 'moderate' symptoms the patient is listed only under the 'moderate' symptoms group

^‡ ^Based on the F test of the joint hypothesis of no differences in the mean quality of life scores across hypoglycemic symptom severity groups

Adjusted linear regression analyses revealed that QOL (EQ-5D VAS) was significantly inversely associated with both the presence of hypoglycemic symptoms (*p *< 0.0001) and the severity of hypoglycemic symptoms after adjusting for other covariates (Table 3). The presence of hypoglycemic symptoms was associated with a reduction 4.33 (*p *< 0.0001) units of the EQ-5D VAS. Relative to those not reporting hypoglycemic symptoms, the reduction in the EQ-5D VAS was 2.68 units (*p *= 0.0039) among those reporting mild symptoms, 6.42 (*p *< 0.0001) among those reporting moderate, and 16.09 (*p *< 0.0001) among those with severe symptoms.

**Table 3:** Factors associated with patients' rating of their health-related quality of life state (EuroQol Visual Analogue Scale) - adjusted linear regression analyses

Variable	Model 1*		**Model 2**^**†**^	
	
	Parameter Estimate	*p*-value	**Parameter Estimate**.	*p*-value
Hypoglycemic Symptoms (yes = 1)	-4.33	< 0.0001	**-**	**-**
Mild Symptoms (yes = 1)	**-**	**-**	-2.68	0.0039
Moderate Symptoms (yes = 1)	**-**	**-**	-6.42	< 0.0001
Severe Symptoms (yes = 1)	**-**	**-**	-16.09	< 0.0001
Age (in years)	-0.07	0.0841	-0.07	0.0917
Female (yes = 1)	-2.65	0.0015	-2.56	0.0021
No Regular Physical Activity (yes = 1)	-2.35	0.0106	-2.06	0.0243
Physical Activity 3-5 Times/Week (yes = 1)	5.08	< 0.0001	5.02	< 0.0001
Weight (in Kg, 0 if missing)	-0.09	< 0.0001	-0.09	0.0002
Missing Weight Indicator (yes = 1)	-11.88	< 0.0001	-11.53	< 0.0001
History of Microvascular Events (yes = 1)	-6.52	0.0191	-6.57	0.0175
History of Cardiovascular Events (yes = 1)	-2.71	0.0042	-2.69	0.0042
HbA_1C _Level	-1.23	0.0011	-1.23	0.0010

Number of Observations	1558		1558	

* The adjusted linear regression reported in model 1 is the result of a backward selection model technique applied on a model including all variables that were significant at *p *≤ 0.20 in univariate analysis. Instead of the hypoglycemic symptom severity effects it contains an indicator for the experience of hypoglycemic symptoms. In addition to the variables reported above, the resulting model included indicators for Spain, France, UK, Finland, and Germany.

† The adjusted linear regression reported in model 1 is the result of a backward selection model technique applied on a model including all variables that were significant at *p *≤ 0.20 in univariate analysis. Instead of the indicator for the experience of hypoglycemic symptoms it contains symptom severity effects with no hypoglycemic symptoms being the reference group. In addition to the variables reported above, the resulting model included indicators for Spain, France, UK, Finland, and Germany.

**Figure 2:**
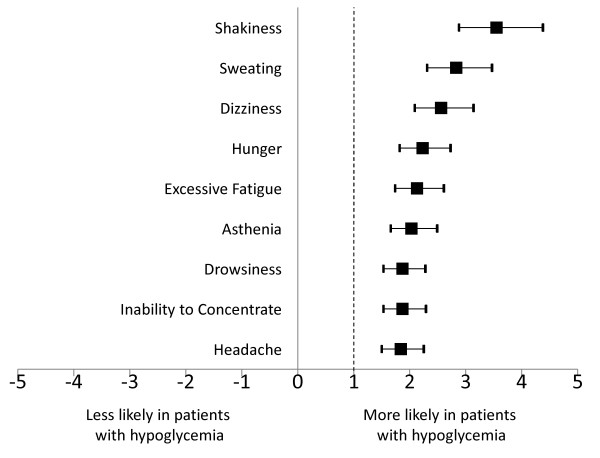
**Patient-reported experience of treatment-related adverse events and experience of hypoglycemic symptoms**. Points (filled boxes) signify odds ratios; error bars signify 95% confidence intervals.

Compared with patients not reporting hypoglycemic symptoms, patients with hypoglycemic symptoms also reported significantly higher rates of each of the treatment-related adverse events evaluated (*p *< 0.0001 for each comparison; not shown). Patients with hypoglycemic symptoms had a more than 3.5-fold increased risk of shakiness (OR, 95% CI 3.55, 2.88-4.38), and an almost 3-fold increased risk of sweating (OR, 95% CI 2.83, 2.31-3.47) (Figure 2). They also had about 2-fold increased risks of excessive fatigue, drowsiness, inability to concentrate, dizziness, hunger, asthenia, and headache.

## Discussion

Hypoglycemia is associated with decreased QOL in European patients with T2DM who are receiving oral antihyperglycemic treatment in usual-care clinical settings. In this study, patients with symptoms of hypoglycemia had significantly lower EQ-5D VAS scores compared with patients without symptoms, indicating worse patient-reported QOL. Increasing hypoglycemic symptom severity was associated inversely with patient QOL. Patients with severe symptoms of hypoglycemia had lower EQ-5D VAS scores compared with patients with moderate, mild, or no symptoms. As might be expected, patients reporting hypoglycemic symptoms reported significantly higher rates of treatment-related shakiness, sweating, excessive fatigue, drowsiness, inability to concentrate, dizziness, hunger, asthenia, and headache.

The association of hypoglycemic symptoms and their severity with patients' perception of health needs to be addressed in conjunction with both diabetes severity and other diabetic complications. Hypoglycemic symptoms may be a manifestation of more intensive treatments in response to more severe diabetes. In addition, more severe diabetes may be associated with the presence of diabetic complications. To examine the association of hypoglycemic symptoms and their severity with patients' perception of health, net of the effect of diabetes severity and diabetic complications, we performed adjusted linear regression analyses controlling for other confounders. The results demonstrated that hypoglycemic symptoms and their severity were independent predictors adversely impacting EQ-5D VAS sores. Measures of diabetes severity (age and level of HbA_1c_) and presence of diabetic complications (history of microvascular events and cardiovascular events) were also significantly associated with patients' perception of health.

While in the pooled analysis both the presence as well as the severity of patient reported hypoglycemic symptoms were associated with EQ-5D VAS, such associations were not uniformly significant across all countries. With the exception of data from Spain, where a relatively large sample did not demonstrate significant associations, the rest of the countries with no significant associations were represented with a small number of observations. This may be one of many reasons for the observed country variations in our analysis.

These results, which demonstrate that both the presence and severity of hypoglycemic symptoms are associated with deleterious effects on QOL in patients with T2DM that are ineffectively managed with metformin, are consistent with and extend previously reported findings. Other groups have also demonstrated that hypoglycemia can erode patient well-being and compromise QOL, as assessed by either the Quality of Well-Being Self-Administered questionnaire in patients with T2DM or both the EQ-5D and the SF-36 in individuals with T1DM or T2DM [11-14]. The current study extended these findings to European patients in usual-care clinical settings who receive oral antihyperglycemic medications. Worsening QOL is also consistent with higher rates of treatment-related adverse events. These results complement earlier findings from the RECAP-DM study that demonstrated a significant inverse association between hypoglycemia and treatment satisfaction, as well as a direct association between hypoglycemia and several barriers to treatment adherence [20].

Hypoglycemic symptoms were based on patient recall of hypoglycemic episodes during the previous year. Hypoglycemic episodes were not verified through measurements of blood glucose levels, neither was there an assessment of the correlation between hypoglycemic symptom severity and blood glucose levels. While impaired awareness of hypoglycemia is more common in patients diagnosed with type-1 diabetes, it may be more prevalent among T2DM patients treated with oral anti-hyperglycemic medications than thought [21,22]. The findings of this study are limited in that they report on the association between subjective, patient reported hypoglycemic symptoms and their severity with patients' health rating. In addition, the availability of only the EQ-5D VAS measure, does not allow consideration of which dimensions capture the consequences of hypoglycemic symptoms on health related quality of life if any at all. Additional studies are needed to confirm these findings and further evaluate the impact of hypoglycemic symptoms on patients' health-related quality of life.

Other possible study limitations include the observational nature of this study, which does not preclude certain potential biases, including selection bias because of the use of a non-probability-based sample of physicians and patients. The study eligibility requirement of at least one HbA_1c _measurement within 12 months may have selected for patients with more intensive diabetes management. Further, the study excluded patients who responded well to metformin monotherapy and relied on self-report to evaluate hypoglycemia and effects on QOL, which may be compromised by, among other factors, focal neurocognitive deficits secondary to glycemic dysregulation. Certain imbalances in baseline comorbidities and other factors could result in confounding by indication in the absence of randomization or propensity score matching.

## Conclusions

This study demonstrated that subjective, patient reported hypoglycemic symptoms are significantly associated with a lower rating for their health related QOL state in patients with T2DM who added sulfonylurea or thiazolidinedione to failing metformin monotherapy in usual-care clinical settings in Europe. Treatments that minimize the risk of and severity of hypoglycemic symptoms while enhancing overall glycemic control hold the promise of promoting superior patient-related outcomes, including QOL, treatment satisfaction, and treatment adherence.

## Competing interests

F Alvarez Guisasola: The author declares that he has no competing interests. D Yin, G Nocea, Y Qiu, and P Mavros are employees of Merck & Co., Inc., the sponsor on this study and analyses.

## Authors' contributions

FAG, GN, YQ were involved in interpreting results, and writing, reviewing and revising report critically for important intellectual content. DY and PM were involved in study design, data acquisition and analysis, interpreting results, and writing, reviewing and revising report critically for important intellectual content. All authors read and approved the final manuscript.
